# Empirically Evaluated Suicide Prevention Program Approaches for Older Adults: A Systematic Review

**DOI:** 10.1093/geroni/igab046.3112

**Published:** 2021-12-17

**Authors:** Vivian Miller, Noelle Fields, Ling Xu, Marta Mercado-Sierra, Marissa Wallace

**Affiliations:** 1 Bowling Green State University, Bowling Green, Ohio, United States; 2 University of Texas at Arlington, Arlington, Texas, United States; 3 Texas A&M Commerce, Commerce, Texas, United States

## Abstract

Suicide is a serious public health concern, particularly for individuals in later life. Studies suggest that greater attention to suicide prevention programs for older adults is needed as well as continued research related to interventions with older adults at risk of attempting suicide. A systematic review of the literature on suicide prevention treatment and effectiveness is fundamental to assessing existing services and developing new programs and practice standards. This systematic review of the literature extends an earlier and well-cited systematic review (1966-2009) by examining articles published between 2009 and 2021 with a focus on what types of empirically evaluated suicide prevention programs effectively prevent and reduce suicidality in older adults. The Preferred Reporting Items for Systematic Reviews and Meta-Analyses guidelines were used to gather the appropriate extant research and improve reporting accuracy. A three-stage review guided the selection of the articles. At stage one, titles were screened, which excluded 284 articles based on the inclusion criteria. Second, after a full review of each abstract, a final 14 articles remained for full-text review. Lastly, three independent researchers reviewed each of the full-text articles, and six articles were excluded. The final sample includes eight articles (N=8). The articles were categorized into three types of programs: 1) primary and home health care, 2) community-based outreach, and 3) counseling. Following a description of the articles, the authors assessed each study using the GRADE rating system. Findings underscore the critical need for evidence-based suicide prevention programs for older adults. Implications for future research are offered.

